# Identification and Characterization of Hypothalamic Alternative Splicing Events and Variants in Ovine Fecundity-Related Genes

**DOI:** 10.3390/ani10112111

**Published:** 2020-11-13

**Authors:** Zhuangbiao Zhang, Jishun Tang, Xiaoyun He, Ran Di, Xiaosheng Zhang, Jinlong Zhang, Wenping Hu, Mingxing Chu

**Affiliations:** 1 Key Laboratory of Animal Genetics and Breeding and Reproduction of Ministry of Agriculture and Rural Affairs, Institute of Animal Science, Chinese Academy of Agricultural Sciences, Beijing 100193, China; zhangzhuangbiao18@163.com (Z.Z.); tjs157@163.com (J.T.); hedayun@sina.cn (X.H.); diran@caas.cn (R.D.); 2 Institute of Animal Husbandry and Veterinary Medicine, Anhui Academy of Agricultural Sciences, Hefei 230031, China; 3 Tianjin Institute of Animal Sciences, Tianjin 300381, China; zhangxs0221@126.com (X.Z.); jlzhang1010@163.com (J.Z.)

**Keywords:** alternative splicing, transcriptomics, proteomics, SNPs, hypothalamus, sheep

## Abstract

**Simple Summary:**

Previous studies revealed that alternative splicing (AS) events and gene variants played key roles in reproduction. However, their location and distribution in hypothalamic fecundity-related genes in sheep without the FecB mutation remain largely unknown. In this study, we performed a correlation analysis of transcriptomics and proteomics, and the results suggested several differentially expressed genes (DEGs)/differentially expressed proteins (DEPs), including galectin 3 (LGALS3), aspartoacylase (ASPA) and transthyretin (TTR), could be candidate genes influencing ovine litter size. Further analysis suggested that AS events, single nucleotide polymorphisms (SNPs) and microRNA (miRNA)-binding sites existed in key DEGs/DEPs, such as ASPA and TTR. This study provides a new insight into ovine and even other mammalian reproduction.

**Abstract:**

Previous studies revealed that alternative splicing (AS) events and gene variants played key roles in reproduction; however, their location and distribution in hypothalamic fecundity-related genes in sheep without the FecB mutation remain largely unknown. Therefore, in this study, we described the hypothalamic AS events and variants in differentially expressed genes (DEGs) in Small Tail Han sheep without the FecB mutation at polytocous sheep in the follicular phase vs. monotocous sheep in the follicular phase (PF vs. MF) and polytocous sheep in the luteal phase vs. monotocous sheep in the luteal phase (PL vs. ML) via an RNA-seq study for the first time. We found 39 DEGs with AS events (AS DEGs) in PF vs. MF, while 42 AS DEGs were identified in PL vs. ML. No DEGs with single nucleotide polymorphisms (SNPs) were observed in PF vs. MF, but five were identified in PL vs. ML. We also performed a correlation analysis of transcriptomics and proteomics, and the results suggested several key DEGs/differentially expressed proteins (DEPs), such as galectin 3 (LGALS3) in PF vs. MF and aspartoacylase (ASPA) and transthyretin (TTR) in PL vs. ML, could be candidate genes influencing ovine litter size. In addition, further analyses suggested that AS events, SNPs and miRNA-binding sites existed in key DEGs/DEPs, such as ASPA and TTR. All in all, this study provides a new insight into ovine and even other mammalian reproduction.

## 1. Introduction

In recent decades, many interesting phenomena have been revealed. The prominent one is the fact that the complexity of diverse organisms is not correlated with the number of genes coding proteins based on genome sequencing, which has gained much attention. Alternative splicing (AS) was then found to be responsible for this difference. AS is a mechanism by which primary transcripts stemming from protein-coding genes could be spliced into distinct isoforms and lead to the generation of diverse variants of proteins [1]. Therefore, comparatively fewer protein-coding genes can generate many proteins with different functions, due to AS events, and maintain the cellular complexity of mammals. The outcomes of complexity caused by AS events could be increasing proteome diversity; introducing the terminal codons responsible for downregulation of mRNA expression; varying the untranslated regions (UTRs) which may influence the binding of non-coding RNAs and RNA stability [2,3,4]. Several methods can be used to detect AS events, such as the analysis of expressed sequence tags or cDNA [1], microarrays technology [5] and recently developed in-depth genome-wide sequencing [6].

AS events play major roles in diverse mammalian physiological activities. Detecting these AS events may, therefore, facilitate a better understanding of the functional specialization of cell types and tissues [7]. Researchers have suggested that AS events were associated with diseases, including a novel biomarker for predicting triple-negative breast cancer [8] and AS-associated genetic variants that increase the risks of bladder cancer in human [9]. Reproduction is a key process for the generation of offspring in mammals. Ongoing research has found close relationships between AS events and reproductive events. Li et al. [10] revealed that AS events found in the testes of Mongolian horses may be a key factor affecting spermatogenesis. A novel splice variant was discovered in *Hydroxysteroid 17-Beta Dehydrogenase 3* genes in the testes of pigs and was highly expressed in Leydig cells, suggesting its key role in male reproductive traits [11]. Efforts were also directed to explore the roles of AS events in female reproduction. Estrogen receptor alpha isoform delta 7 (ERΔ7) generated by AS events in the female myometrium functioned as a modulator of myometrial quiescence, which may facilitate fetal development [12]. Importantly, Miao et al. [13] uncovered many AS events associated with fecundity in different sheep breeds via ovarian transcriptomic analysis, suggesting the key roles of AS events in ovine reproduction.

Single nucleotide polymorphisms (SNPs) and indels in genes can significantly influence gene and protein functions. FecB, one of the most famous genes influencing ovine fecundity, was a point mutation (from A to G) that occurred at base 746 of the *bone morphogenetic protein receptor*, *type 1B* gene [14]. Most importantly, FecB has a dosage effect, which indicated that ewes with one copy of the FecB mutation could increase litter size by 1, while two copies of this mutations could significantly increase litter size by 1.5 [15]. Further studies suggested that SNPs in fecundity genes, such as the *growth differentiation factor 9* [16], *bone morphogenetic protein 15* (BMP15) [17], *BMP2*, *BMP7* [18], *the NLR family pyrin domain-containing 5* (*NLRP5*) and *NLRP9* [19], were highly associated with litter size in sheep. Regarding indels, RNA-seq can be an effective method to detect indels as demonstrated by different bioinformatics strategies in many fields such as clinical decision-making [20], and many studies also found their key roles in litter size. The 11-bp insertion variant of intron 22 in *DNA methyltransferase 3β* was highly associated with litter size in goats [21]. Three indels were detected in *down syndrome cell adhesion molecule like 1*, and further association analysis suggested that these indels were highly associated with litter size in goats [22]. All in all, RNA-seq can be used as a reliable and effective method to detect AS events, SNPs and indels.

With the development of sequencing technology, RNA-sequencing has widely been used to identify key genes and non-coding RNAs affecting complex traits such as reproduction. Zou et al. [23] identified 289 differentially expressed genes (DEGs) from uniparous and multiple goats, and further analysis indicated that *CD36*, *TNFAIP6*, *CYP11A1*, *SERPINA5* and *PTGFR* may be the candidate genes associated with fecundity. The pituitary is a key reproductive organ controlling hormone activities; previous pituitary transcriptomics studies identified *SMAD2*, *NMB* and *EFNB3* as the key genes affecting ovine prolificacy in different fecundity sheep [24]. Our previous study also indicated that *GNRH1*, *PRL*, *GH*, *TRH* and *TTR* may regulate the gonadotropin-releasing hormone (GnRH) activities in the hypothalamus [25]. Proteins are the real embodiment of gene functions, and therefore, identifying candidate genes from the translational level can be more biologically informative than from the transcriptional level. Tang et al. [26] uncovered several fecundity proteins, such as StAR and HSD3B, in sheep with different fecundities by ovarian proteomics. Our previous hypothalamic proteomics studies also suggested that (growth hormone) GH, insulin-like growth factor 1 receptor (IGF1R) and Transthyretin (TTR) played key roles in GnRH release [27]. Importantly, transcription and translation are under dynamic changes and are different processes controlled by many factors. Therefore, combining transcriptomics and proteomics data can be an effective way to identify key genes associated with fecundity.

Sheep are one of the animals whose reproduction was mainly controlled by hormones activities, and one of the key roles of the hypothalamus is initiating reproduction by releasing GnRH. Therefore, in this study, we combined the hypothalamic transcriptomic and proteomic data to identify key genes at transcriptional and translational levels and to try to explore the AS events and variants associated with reproduction occurred in hypothalami of sheep with different fecundities via hypothalamic RNA-seq. Our study may provide new insights and references for ovine reproduction.

## 2. Materials and Methods

### 2.1. Animal Selection and Samples Preparation

All experimental procedures involving animals used in this study were approved by the Science Research Department (in charge of animal welfare issues) of the Institute of Animal Science, Chinese Academy of Agricultural Sciences (IAS-CAAS; Beijing, China). In addition, ethics approval was granted by the animal ethics committee of IAS-CAAS.

First, Small Tail Han sheep (n = 890) were genotyped by the TaqMan probe [28], and 12 ewes without the FecB mutation were selected and grouped into polytocous (ovulation and litter size ≥ 2, n = 6) and monotocous populations (ovulation and litter size = 1, n = 6) for estrus synchronization via the controlled internal drug releasing device (CIDR) method [25]. All 12 selected ewes were treated with CIDR for 12 days, and three polytocous and three monotocous ewes were slaughtered with euthanasia at 45–48 h (follicular phase) after removing CIDR and the whole hypothalami were immediately collected and stored at −80 °C. The remaining 6 ewes were slaughtered on day nine (luteal phase) after removing CIDR and the whole hypothalami were immediately collected and stored at −80 °C. All selected ewes were divided into polytocous sheep at the follicular phase (PF), polytocous sheep in the luteal phase (PL), monotocous sheep in the follicular phase (MF) and monotocous sheep in the luteal phase (ML). All of the ewes used in this study were reared in the sheep farm of the Tianjin Institute of Animal Sciences with the same feeding regime.

### 2.2. Total RNA Extraction and Sequencing

Total RNA was extracted from 12 hypothalamic tissues collected before using TRIzol Reagent (Invitrogen, Carlsbad, CA, USA). To obtained high-quality RNA, the concentration, purity and integrity of total RNA were measured and assessed by the Kaiao K5500 spectrophotometer (Kaiao Technology Development Co. Ltd., Beijing, China), the RNA Nano 6000 Assay Kit of the Agilent Bioanalyzer 2100 System (Agilent Technologies, Santa Rosa, CA, USA) and 1% agarose electrophoresis, respectively. Each sample shared 3 μg total RNA for library construction, and rRNA was eliminated by Ribo-Zero™ Gold Kits (Epicentre, Madison, WI, USA). The library was built according to the operating instructions of the NEB Next Ultra Directional RNA Library Prep Kit for Illumina (NEB, Ipswich, MA, USA); the detailed method regarding library construction has been described elsewhere [25]. Finally, the constructed library was sequenced by Illumina HiSeq 2500.

### 2.3. Filtering Raw Data and Mapping

The sequencing results were presented originally as an image data file, which was then transferred into raw reads by identifying bases via bcl2fastq software. The raw data were filtered to obtain clear data under the following criteria: eliminating raw reads of adapter contamination; removing low-quality reads (bases with Q ≤ 19 in reads account for more than 15% of the total bases); removing the raw reads where the unknown base number was more than 5%. To map the clean reads against the ovine genome, we downloaded the reference genome and annotation files from ENSEMBL (http://www.ensembl.org/index.html), and then the obtained high-quality clean data were mapped to the ovine genome (Oar v.3.1) via HiSAT2 (--rna-strandness RF --dta–t–p 4) [29].

### 2.4. Transcriptome Assembly and Differential Expression Analysis

To obtain integrated transcripts, StringTie (-G ref.gtf–rf–l) [30] was applied to assemble transcripts based on mapping results. The expression level of identified transcripts was shown as Fragments Per Kilobase of transcript per Million mapped reads (FPKM) values. First, we calculated the read numbers mapping to the gene by HTSeq [31], then we estimated the expression level of each gene and obtained FPKM values [32].

We used DESeq (method = ‘per-condition’) [33] to conduct differential expression analysis. We set fold change >1.6 and *p* < 0.05 as the threshold of DEGs.

### 2.5. Identification of AS Events, SNPs and Indels

To identify AS events, Asprofile [34] was applied to calculate and classify AS events and the FPKM tool in Asprofile software was then used to obtain the structural characteristics of AS events. Finally, the identified AS events were annotated by the reference genome (Oar v.3.1). To analyze the variants in samples, SAMtools mpileup [35] was implemented to obtain bcf files(-ugf); then, BCFtools (-d 3 –D 50) was used to screen variants for quality and give vcf files.

### 2.6. Correlation Analysis between Proteomics and Transcriptomics

The proteome data have been previously described elsewhere [27]. Spearman’s correlation coefficient was used to assess the correlation between proteomes and transcriptomes. Correlative analysis was performed between proteomes and transcriptomes for all groups, including the correlative analysis of differentially expressed proteins (DEPs) and DEGs, where both showed the same expression trend (DEPs_DEGs_SameTrend); the correlative analysis of DEPs and DEGs, where both showed the opposite expression trend (DEPs_DEGs_Opposite); correlative analysis of DEPs and non-DEGs (DEPs_NDEGs); the correlative analysis of non-DEPs and DEGs (NDEPs_DEGs); the correlative analysis of non-DEPs and non-DEGs (NDEPs_NDEGs).

### 2.7. Gene Ontology (GO) and Kyoto Encyclopedia of Genes and Genomes (KEGG) Enrichment Analysis

To perform gene ontology (GO) and Kyoto Encyclopedia of Genes and Genomes (KEGG) enrichment analysis, the KOBAS 3.0 [36] online software (http://kobas.cbi.pku.edu.cn/kobas3) was used to conduct GO and KEGG enrichment analysis, where *p* < 0.05 was regarded as significant enrichment. First, we input genes/proteins, and then we selected species of sheep and chose GO and KEGG analysis, until, finally, we could obtain the enrichment results.

## 3. Results

### 3.1. mRNAs Profiling

To fully characterize the expression pattern of hypothalamic mRNAs, we performed hypothalamic RNA-seq analyses. The results suggested that the mapping rate of all samples used in this study was more than 91% (Table 1), which suggested that the data obtained by RNA-seq had a higher quality. Further analysis suggested that 21,221 genes were recognized, and chromosome 3 contains the most genes, followed by chromosomes 1 and 2. In addition, based on the threshold of fold change >1.6 and *p* < 0.05, we identified 172 DEGs in PF vs. MF (Table S1), where 79 genes were upregulated while 93 genes were downregulated. Furthermore, 235 DEGs were detected in PL vs. ML (Table S1), where 90 genes were upregulated while 145 genes were downregulated. Other details regarding mRNA profiling have been described elsewhere [25].

### 3.2. Identification and Characterization of AS Events

In this study, we identified AS events using Asprofile from 12 hypothalamic samples. We identified AS events of 12 types in total (Table 2, Table S2), and most AS events were found in the transcription start site (TSS) and transcription terminal site (TTS). AS events of the TSS type accounted for nearly 40% of all detected AS events, followed by AS events of the TTS type, and AS events of the TSS and TTS types comprise about 70% of all identified AS events.

### 3.3. The AS Events Found in DEGs

**Figure 1:**
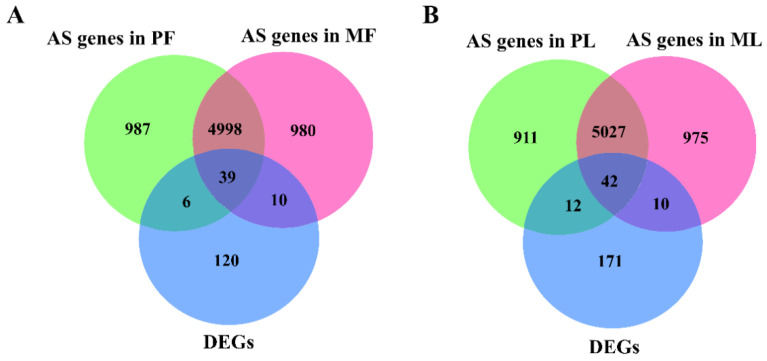
The identified genes with AS events (AS genes) in polytocous sheep at the follicular phase (PF), polytocous sheep in the luteal phase (PL), monotocous sheep in the follicular phase (MF) and monotocous sheep in the luteal phase (ML). (**A**) The identified AS genes and their intersections with differentially expressed genes (DEGs) in PF and MF; (**B**) the identified AS genes and their intersections with DEGs in PL and ML.

**Figure 2:**
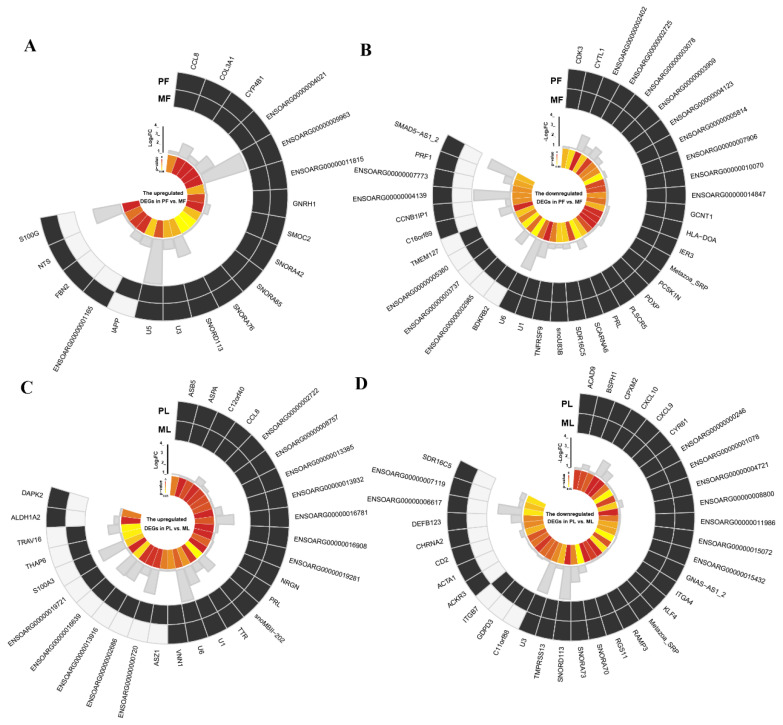
The detailed information of identified differentially expressed AS genes (DE AS genes) in PF vs. MF and PL vs. ML. (**A**) The detailed information of identified upregulated DE AS genes in PF vs. MF; (**B**) the detailed information of identified downregulated DE AS genes in PF vs. MF; (**C**) the detailed information of identified upregulated DE AS genes in PL vs. ML; (**D**) the detailed information of identified downregulated DE AS genes in PL vs. ML.

To screen key AS events affecting reproduction, we firstly mapped these identified AS events to ovine genomes; then, these mapped genes were screened from the databases of DEGs identified by RNA-seq. In PF, we identified 6030 genes with AS events (AS genes) of 12 types, while 6027 genes with AS events in MF were detected. Additionally, 5037 genes with AS events were shared between PF and MF, while 39 were shared between those 5037 genes and DEGs (Figure 1A, Table S3), and details of differentially expressed AS genes (DE AS genes), including fold change and up-/down-regulation in PF vs. MF, were shown in Figure 2A,B. In PL, we identified 5992 genes with AS events of 12 types in total, while 6054 genes with AS events in ML were detected. Furthermore, 5069 genes with AS events were shared between PL and ML, while 42 were shared between these 5069 genes and DEGs (Figure 1B, Table S4), and details of DE AS genes, including fold change and up-/down-regulation in PL vs. ML, were shown in Figure 2C,D. Combined, we found 39 DE AS genes in PF vs. MF and 42 DE AS genes in PL vs. ML.

### 3.4. Functional Enrichment Analysis of DE AS Genes

**Figure 3:**
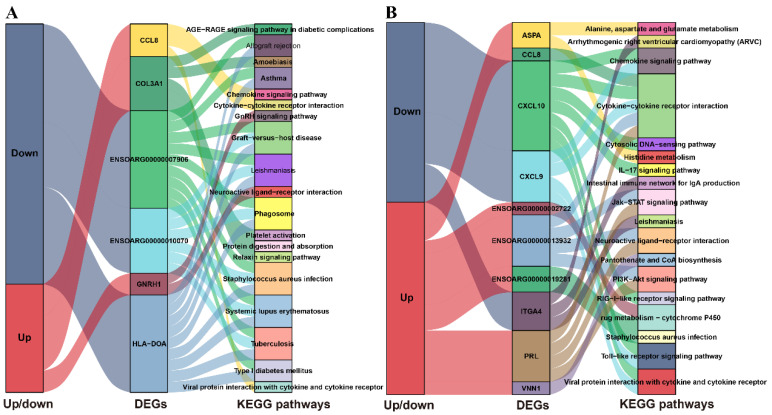
The top ten enriched Kyoto Encyclopedia of Genes and Genomes (KEGG) pathways in up/downregulated DE AS genes in PF vs. MF (**A**) and in PL vs. ML (**B**).

To fully understand the functions of the identified DE AS genes, we performed functional enrichment analysis of DE AS genes via KOBAS 3.0 online software. First, we divided all DE AS genes into upregulated and downregulated groups, then KEGG pathways enrichment analysis was conducted based on the above groups. We selected the top ten KEGG pathways in each group to plot Figure 3. The results (Figure 3A) suggested that the most enriched pathway of upregulated DE AS genes in PF vs. MF was viral protein interaction with cytokine and cytokine receptor, while the most enriched pathway by down-regulated DE AS genes in PF vs. MF was *Staphylococcus aureus* infection. Further analysis found that most common pathways within the top 10 pathways enriched by DE AS genes were related to diseases. Notably, a pathway named the GnRH-signaling pathway was also highly enriched by upregulated DE AS genes in PF vs. MF and was associated with reproduction. In PL vs. ML (Figure 3B), the most common pathway enriched by upregulated DE AS genes was cytokine–cytokine receptor interaction, while the most enriched pathway in down-regulated DE AS genes was viral protein interaction with cytokine and cytokine receptor. Furthermore, other pathways associated with reproduction, such as the Jak-STAT signaling pathway and PI3K-Akt signaling pathway, were also within the top ten enriched pathways. However, we failed to enrich the GO terms due to the limited number of DE AS genes.

### 3.5. Interaction Analysis of DE AS Genes with microRNA (miRNA)

**Figure 4:**
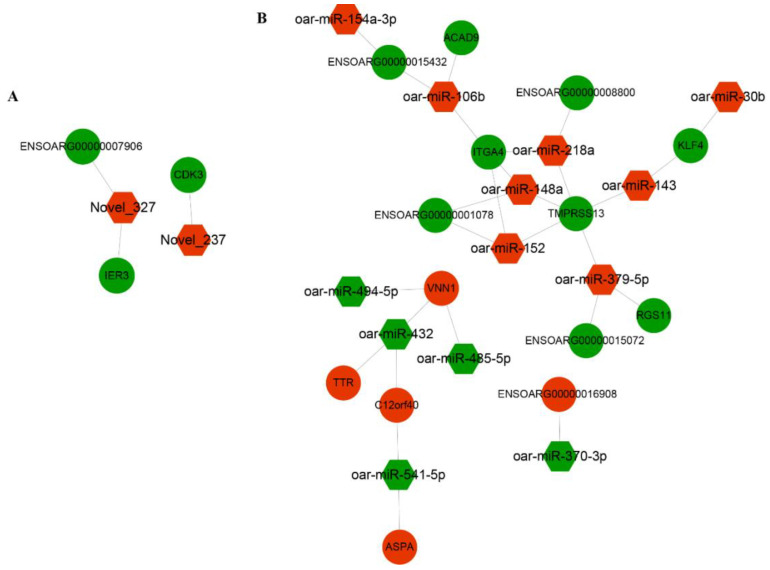
The interaction analysis of DE AS genes with DE microRNA in PF vs. MF (**A**) and in PL vs. ML (**B**).

A previous study reported that microRNAs (miRNAs) could facilitate the AS events of genes [37]. Therefore, we conducted DE AS gene-miRNAs analysis using DE AS genes and DE miRNA to understand the factors affecting AS events. Notably, the selected interactome of DE AS genes–miRNAs showed reverse regulatory relationships. The results of miRNA analysis have been previously described [25]. The interactive analysis suggested that two novel DE miRNAs targeted three DE AS genes in PF vs. MF (Figure 4A), while 14 DE AS genes were targeted by 13 DE miRNAs in PL vs. ML (Figure 4B), and most miRNAs targeted more than one DE AS gene. For example, *TMPRMM13* was co-regulated by five DE miRNAs.

### 3.6. The SNPs and Indels Analysis of DEGs

**Figure 5:**
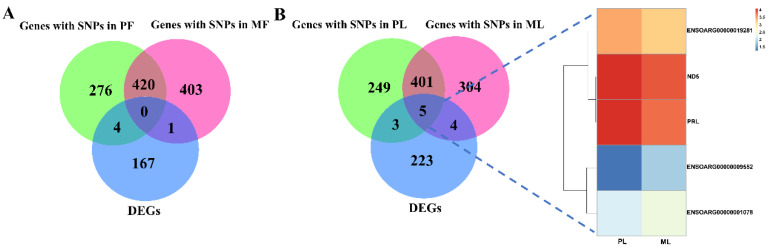
The identified genes with single nucleotide polymorphisms (SNPs) in PF, PL, MF and ML. (**A**) The identified genes with SNPs and their intersections with DEGs in PF and MF; (**B**) the identified genes with SNPs and their intersections with DEGs in PL and ML; five key DEGs with SNPs are also shown by heat map.

To identify SNPs and indels distributed in the hypothalami of ewes without the FecB mutation, variants analysis was performed using SAMtools software. The mapped genes with SNPs were screened from the databases of DEGs identified by RNA-seq (Table S5). In PF, we identified 700 genes with SNPs representing synonymous mutation and missense mutations in total, while 824 genes with SNPs in MF were detected. Furthermore, 420 genes with SNPs were shared between PF and MF, while none were in common between those 420 genes and DEGs (Figure 5A, Table S6). In PL, we identified 658 genes with SNPs representing synonymous mutation and missense mutations in total, while 714 genes with SNPs in ML were detected. In addition, 406 genes with SNPs were shared between PL and ML, while five were in common between those 406 genes and DEGs (Figure 5B, Table S7). The key five DEGs with SNPs are also shown with a heat map (Figure 5B).

**Figure 6:**
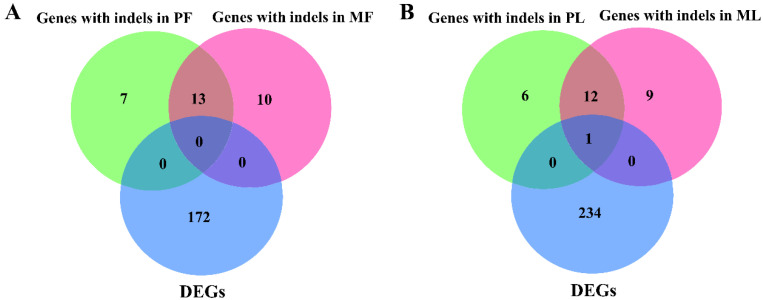
The identified genes with indels in PF, PL, MF and ML. (**A**) The identified genes with indels and their intersections with DEGs in PF and MF, (**B**) The identified genes with indels and their intersections with DEGs in PL and ML.

Indels were also detected in this study. The mapped genes were screened from the databases of DEGs identified by RNA-seq (Table S8). In PF, we identified 20 genes with indels, while 23 genes with indels were detected in MF. The 13 genes with indels were shared between PF and MF. However, none were in common between those 13 genes and DEGs (Figure 6A, Table S9). In PL, we identified 19 genes with indels, while 22 genes with indels in ML were detected. The 13 genes with indels were shared between PL and ML, while one (ENSOARG00000001078) was in common between these 13 genes and DEGs (Figure 6B, Table S10).

### 3.7. Correlation Analysis of the Proteomics and Transcriptomics

**Figure 7:**
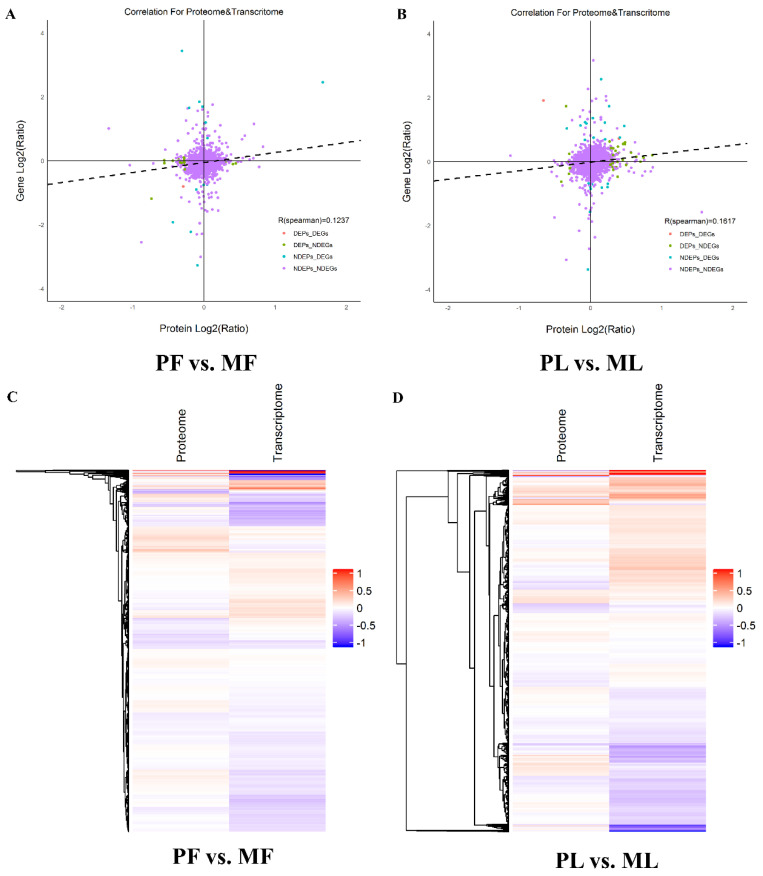
Correlative scatter plots of identified proteins and genes. (**A**) Correlative scatter plots of identified proteins and genes in PF vs. MF. (**B**) Correlative scatter plots of identified proteins and genes in PL vs. ML. (**C**) The heat map analysis of correlative proteins and genes in PF vs. MF. (**D**) The heat map analysis of correlative proteins and genes in PL vs. ML.

To precisely pinpoint the candidate genes associated with litter size in sheep, we combined proteomic and transcriptomic data to identify the key fecundity DEGs. Based on our previous study [26], we identified 22 DEPs in PF vs. MF, where 5 DEPs were upregulated and 17 DEPs were downregulated, and 39 DEPs in PL vs. ML, where 31 DEPs were upregulated and 8 DEPs were downregulated (Table S11). We also assessed the correlation between proteomics and transcriptomics with Spearman’s correlation coefficient (R). The results suggested the R in PF vs. MF was 0.1237 (Figure 7A), while the R in PL vs. ML was 0.1617 (Figure 7B). The correlative proteins and genes in PF vs. MF (Figure 7C) and PL vs. ML (Figure 7D) are also shown in the form of a heat map. The results indicated that some correlative proteins and genes showed the same expression trend, while some of them showed opposite expression trends.

**Figure 8:**
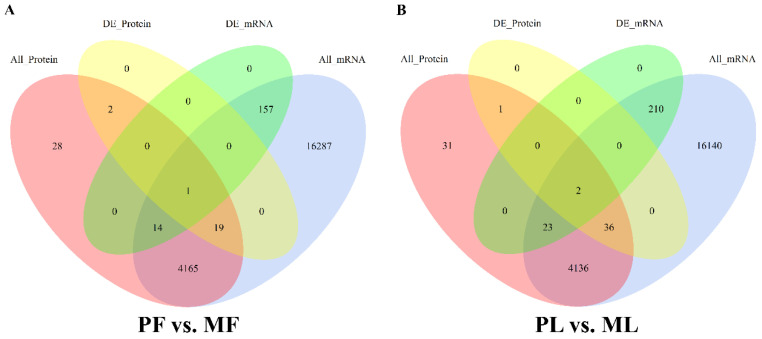
Venn diagram of the correlation between proteomics and transcriptomics in PF vs. MF (**A**) and PL vs. ML (**B**).

We also plotted the Venn diagram to show the details of DEGs and DEPs. In total, 4241 proteins and 20,643 genes in PF vs. MF were used to conduct a correlation analysis (Figure 8A). Only galectin 3 (LGALS3) was shared between DEPs and DEGs, while 14 were shared between non-DEPs and DEGs, 19 were shared between DEPs and non-DEGs and 4165 were in common between non-DEPs and non-DEGs. In total, 4241 proteins and 20,547 genes in PL vs. ML were used to conduct a correlation analysis (Figure 8B). Two genes/proteins, aspartoacylase (ASPA) and transthyretin (TTR), were shared between DEPs and DEGs, while 23 were shared between non-DEPs and DEGs, 36 were shared between DEPs and non-DEGs and 4136 were in common between non-DEPs and non-DEGs.

**Figure 9:**
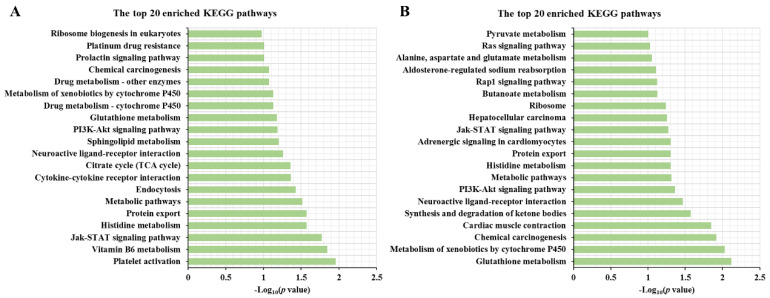
The top 20 KEGG pathways enriched by key genes/proteins in PF vs. MF (**A**) and PL vs. ML (**B**).

To understand the roles of key genes or proteins, we performed KEGG pathways analysis in PF vs. MF and PL vs. ML using the genes/proteins including the intersection of DEPs and DEGs, the intersection of non-DEPs and DEGs and the intersection of DEPs and non-DEGs via KOBAS online software. The enrichment results suggested that the most enriched pathway in PF vs. MF (Figure 9A) was platelet activation, and other fecundity-related pathways, such as the Jak-STAT signaling and PI3K-Akt signaling pathways, were also enriched. The most enriched pathway in PL vs. ML (Figure 9B) was glutathione metabolism. Interestingly, other fecundity-related pathways, such as the Jak-STAT signaling pathway and the PI3K-Akt signaling pathway, were also enriched. In addition, some metabolism-related pathways, such as metabolic pathways and butanoate metabolism, also played important roles in the luteal phase of sheep.

## 4. Discussion

In this study, we identified AS events, SNPs and indels from ovine hypothalami using RNA-seq. RNA-seq is an effective tool to detect and explore such events. Popovitchenko et al. [38] found that an AS event identified by RNA-seq at the 5′ UTR of Elavl4 in mouse was a key factor determining neuronal development. During pregnancy, many AS events identified by RNA-seq also proved to be necessary for rats [39]. RNA-seq, as a widely used technology to quantify gene expression, was also used for SNP detection. Previous research explored several potential point mutations associated with pig production via RNA-seq, which were also validated by association analysis [40]. Fischer et al. [41] uncovered many SNPs in fecundity genes in pigs by RNA-seq study, and they also verified the existence of identified SNPs by Sanger sequencing. There are two protocols that were used to extract total RNA, including Qiagen and TRIzol, and two approaches that were also used for RNA-seq, including poly(A)+ and RiboZero. Therefore, different combinations could have different effects. The reads mapping to intronic regions of genome using TRIzol were higher than using Qiagen, and most of them were attributed to hnRNA found in the nucleus, which was much more obvious using the RiboZero method than using poly(A)+. In addition, the expression level of protein-coding genes identified using poly(A)+ RNA-seq may be higher than that from using RiboZero, and the data obtained from RiboZero may be unclear if the power to detect AS events is not enough [42]. Given the time and process of AS events, the poly(A)+-based RNA-seq may be a more powerful way to detect AS events in future studies.

In this study, many DEGs with alternative splicing were uncovered and several of them have potential roles in reproduction. *Gonadotropin releasing hormone 1* (*GNRH1*) was detected as an upregulated DEG in PF vs. MF and was enriched in the GnRH signaling pathway. many researches have reported its key roles in hormone activities and litter size. It is well known that GNRH1 in the hypothalamus is indispensable for release of follicle-stimulating hormone and luteinizing hormone [43], while both hormones are crucial for follicular development. In addition, GNRH1 is functionally conserved across a wide range of species and is a decapeptide connecting the brain and with the peripheral reproductive system [44]. A further study suggested GNRH1 can be modulated to influence litter size in mice [45]. *GNRH1* was also validated as a candidate gene affecting litter size in goats [46]. Importantly, in our study, the expression level of GNRH1 in PF was higher than that of MF, which may further result in the concentration difference of GnRH and affect ovulation rate. Interestingly, *GNRH1* was also predicted to be a DE AS gene, and AS events may influence the concentration of hypothalamic GnRH responsible for initiating reproduction.

Prolactin (PRL) was recognized as a key factor modulating GnRH activities and was enriched in the Jak-STAT signaling [47] and PI3K-Akt signaling pathways [48], both of which were reported to participate in GnRH activities. A previous study revealed that PRL in the hypothalami of mice could inhibit LH release, probably by involving GnRH release, and kisspeptin neurons seemed to be the target of PRL-inhibiting GnRH neurons [49]. In our data, PRL was found downregulated in PF vs. MF, which was consistent with the results uncovered in the mice mentioned above. Interestingly, *PRL* was also an upregulated DEG in PL vs. ML and, considering the key prohibited role of E2 on GnRH release in the luteal phase [50], we speculate that PRL and E2 may cooperate to inhibit the GnRH activities. Interestingly, *PRL* was also predicted to be a DE AS gene, and several SNPs were also detected. All of these possible genetic variants may have potential in influencing ovine reproduction.

Correlation analysis of proteomics and transcriptomics uncovered several key DEGs/DEPs, such as LGALS3 in PF vs. MF and ASPA and TTR in PL vs. ML. The proteomics and transcriptomics analysis indicated that LGALS3 was downregulated at both transcriptional and translational levels. Previous research reported that LGALS3 was highly expressed in multiple nerve nuclei of the hypothalamus in rats, such as the paraventricular hypothalamic nucleus, arcuate hypothalamic nucleus and supraoptic nucleus [51]. All these nerve nuclei, highly expressed by LGALS3, are key components of the hypothalamic–pituitary–gonadal axis [52] and can significantly affect the reproductive activities of animals. ASPA was a key downregulated DEP and DEG screened by proteomics and transcriptomics studies. The female mouse with ASPA deficiency could reduce the pups by about 50% compared to a knockout (KO)/+ female mouse. In addition, female mice with ASPA deficiency could lead to 7% death of pups, which may be caused by damaging the central nervous system [53]. Interestingly, in our study, ASPA was a key DEP and DEG in the ovine hypothalamus, which is a crucial part of the central nervous system. Importantly, the log2 (fold change) of ASPA in proteomic data was 0.40, while the log2 (fold change) of ASPA in transcriptomic was 0.72, and considering the facts that *ASPA* was a DE AS gene in PL vs. ML and was also regulated by oar-miR-541-5p, we speculate that AS events and miRNA binding may affect the expression of ASPA. Combined, we speculate that the ASPA discovered in the hypothalamus functions as a regulator controlling litter size in sheep.

Transthyretin (TTR) may also modulate GnRH activities directly or indirectly. Progesterone in the luteal phase prohibits GnRH release, and progesterone in rats was proved to increase the expression of TTR both in vivo and in vitro [54]. A previous study suggested that TTR could influence the expression level of insulin-like growth factor 1 receptor (IGF1R) by inducing the nuclear translocation of IGF1R [55]. In addition, IGF1 in female rats was also reported to induce GnRH release by activating the expression of *KISS-1* gene [56]. Therefore, the expression change of IGF1R may finally influence the effects of IGF1 on GnRH activities. Therefore, TTR played key roles in GnRH activities. Importantly, TTR was upregulated DEG in PL vs. ML, while it was downregulated DEP in PL vs. ML. This phenomenon seems to be difficult to understand, but it should be noted that *TTR* was also a DE AS gene in PL vs. ML and was also regulated by oar-miR-432. AS events and miRNA binding may cause the opposite expression trend of TTR in the luteal phase in sheep in between transcriptional and translational levels.

## 5. Conclusions

In this study, we have, for the first time, identified several key hypothalamic DEGs/DEPs, such as LGALS3 in PF vs. MF and ASPA and TTR in PL vs. ML, by combining proteomics and transcriptomics, and all of these DEGs/DEPs may be candidate genes influencing ovine litter size. In addition, further analyses suggested that AS events, SNPs and miRNA-binding sites existed in key DEGs/DEPs, such as ASPA and TTR. This study enhanced our understanding for mammalian reproduction and provided insights into ovine fecundity.

## Figures and Tables

**Table 1:** Summary of the filter and mapping of hypothalamic mRNA sequencing data.

Sample	Raw Base	Raw Reads	Clean Base	Clean Reads	Q30 (%)	Mapping Rate (%)
PF_H1	19,170,842,700	127,805,618	18,381,864,300	122,545,762	91.65	91.76
PF_H2	18,659,879,100	124,399,194	17,994,065,700	119,960,438	93.22	92.80
PF_H3	19,849,439,400	132,329,596	18,974,757,300	126,498,382	91.99	92.22
PL_H1	19,326,903,300	128,846,022	18,789,727,800	125,264,852	93.80	92.81
PL_H2	15,196,164,900	101,307,766	14,538,760,200	96,925,068	92.87	92.99
PL_H3	20,028,519,600	133,523,464	19,408,391,100	129,389,274	93.78	92.78
MF_H1	18,753,972,900	125,026,486	18,088,311,900	120,588,746	93.91	92.45
MF_H2	19,560,182,400	130,401,216	18,550,470,900	123,669,806	92.84	92.40
MF_H3	19,950,184,500	133,001,230	19,076,879,400	127,179,196	93.80	92.23
ML_H1	19,028,904,900	126,859,366	18,356,811,000	122,378,740	94.39	92.77
ML_H2	19,034,574,000	126,897,160	18,236,273,700	121,575,158	93.83	92.67
ML_H3	19,262,332,500	128,415,550	18,641,870,100	124,279,134	94.15	92.20

**Table 2:** The hypothalamic alternative splicing (AS) events identified by RNA-seq.

AS_type	PF_H1	PF_H2	PF_H3	PL_H1	PL_H2	PL_H3	MF_H1	MF_H2	MF_H3	ML_H1	ML_H2	ML_H3
AE	3.76%	3.34%	3.46%	3.50%	3.38%	3.54%	3.58%	3.63%	3.89%	3.44%	3.62%	3.78%
XAE	2.90%	3.05%	3.07%	2.70%	2.72%	2.78%	3.12%	3.10%	2.84%	3.07%	3.10%	2.77%
IR	7.15%	7.70%	7.28%	6.46%	7.04%	6.66%	7.68%	7.71%	6.47%	7.47%	7.36%	6.52%
MIR	1.72%	2.20%	1.99%	1.55%	1.76%	1.71%	2.19%	2.12%	1.66%	2.08%	2.18%	1.66%
XIR	2.66%	3.02%	2.83%	2.64%	2.67%	2.64%	3.04%	3.10%	2.38%	3.31%	2.96%	2.52%
TTS	32.80%	33.00%	33.19%	33.80%	34.09%	33.59%	32.88%	32.40%	32.99%	33.08%	32.72%	33.59%
XMIR	0.44%	0.57%	0.51%	0.35%	0.44%	0.43%	0.47%	0.66%	0.38%	0.54%	0.48%	0.44%
SKIP	7.39%	6.10%	6.47%	6.89%	6.47%	6.96%	6.51%	6.73%	7.62%	6.21%	6.88%	7.25%
MSKIP	1.33%	1.06%	1.19%	1.31%	1.12%	1.33%	1.12%	1.26%	1.56%	1.14%	1.21%	1.36%
XMSKIP	0.44%	0.39%	0.33%	0.41%	0.29%	0.39%	0.38%	0.43%	0.49%	0.39%	0.41%	0.37%
XSKIP	2.11%	1.91%	1.89%	2.03%	1.67%	2.03%	1.78%	1.98%	2.17%	1.86%	2.02%	2.14%
TSS	37.33%	37.66%	37.78%	38.35%	38.35%	37.94%	37.24%	36.87%	37.57%	37.41%	37.06%	37.60%

Notes: AE: Alternative exon; XAE: Approximate AE (5′, 3′ or both); IR: Intron retention; MIR: Multi-IR; XIR: Approximate IR; TTS: Transcription terminal site; XMIR: Approximate MIR; SKIP: Skipped exon; MSKIP: Multi-SKIP; XMSKIP: Approximate MSKIP; XSKIP: Approximate SKIP; TSS: Transcription start site.

## Supplementary Materials

The following are available online at https://www.mdpi.com/2076-2615/10/11/2111/s1, Table S1: The overall genes expressed in polytocous sheep in the follicular phase versus monotocous sheep in the follicular phase (PF vs. MF) and polytocous sheep in the luteal phase versus monotocous sheep in the luteal phase (PL vs. ML), where red and green represent up- and down-regulation respectively; Table S2: The AS event type, location and event number found in genes identified from ovine hypothalami; Table S3: The identified genes with AS events (AS genes) in PF and MF and their intersections with DEGs in PF vs. MF; Table S4: The identified genes with AS events (AS genes) in PL and ML and their intersections with DEGs in PL vs. ML; Table S5: The SNP location and base changes identified from ovine hypothalami; Table S6: The identified genes with SNPs in PF and MF and their intersections with DEGs in PF vs. MF; Table S7: The identified genes with SNPs in PL and ML and their intersections with DEGs in PL vs. ML; Table S8: The indel location and base changes identified from ovine hypothalami; Table S9: The identified genes with indels in PF and MF and their intersections with DEGs in PF vs. MF; Table S10: The identified genes with indels in PL and ML and their intersections with DEGs in PL vs. ML; Table S11: The differentially expressed proteins (DEPs) identified from PF vs. MF and PL vs. ML, where red and green represent up- and down-regulation, respectively.
